# Treatment Outcome and Associated Factors among Tuberculosis Patients in Debre Tabor, Northwestern Ethiopia: A Retrospective Study

**DOI:** 10.1155/2016/1354356

**Published:** 2016-08-15

**Authors:** Addisu Melese, Balew Zeleke, Biniam Ewnete

**Affiliations:** ^1^Department of Medical Laboratory Science, College of Health Sciences, Debre Tabor University, P.O. Box 272, Debre Tabor, Ethiopia; ^2^Department of Nursing, College of Medicine and Health Science, Bahir Dar University, Bahir Dar, Ethiopia; ^3^Department of Medicine, College of Health Sciences, Debre Tabor University, P.O. Box 272, Debre Tabor, Ethiopia

## Abstract

*Background*. Assessing the outcomes of tuberculosis (TB) treatment is an important indicator for evaluation of the effectiveness of tuberculosis control programs. In Ethiopia, directly observed treatment short course (DOTS) was included in the national tuberculosis control program as a strategy but little is known about its effectiveness in the study area. Therefore, this study was aimed at assessing the treatment outcomes of TB patients and associated factors in Debre Tabor, northwest Ethiopia.* Methods.* A retrospective study was conducted among TB patients for the period from May 2008 to April 2013 at Debre Tabor Health Center, northwest Ethiopia. Data were entered and analyzed using SPSS version 20.0. Descriptive statistics were used to generate frequency tables and figures. Logistic regressions were used to identify factors associated with treatment outcomes at *P* value ≤ 0.05.* Results.* Out of 339 patients (197 males and 142 females) registered for antituberculosis treatment in Debre Tabor Health Center, only 303 patients were included in the treatment outcome analysis and 87.1% had successful treatment outcome while 12.9% had unsuccessful treatment outcome. In the multivariate logistic regression analysis, the odds of successful treatment outcome were higher among patients ≥45 years of age (AOR = 3.807, 95% CI: 1.155–12.544) and lower among females (AOR = 0.347, 95% CI: 0.132–0.917), rural residents (AOR = 0.342, 95% CI: 0.118–0.986), and negative smear result at the second month of treatment (AOR = 0.056, 95% CI: 0.005–0.577) as compared to their counterparts.* Conclusion.* The treatment outcome of all forms of tuberculosis patients in Debre Tabor health center was satisfactory as expected from effective implementation of DOTS. Although the observed successful treatment outcome was in agreement with the national target, follow-up of patients during the course of treatment to trace the treatment outcomes of transferred-out patients and assessment of other potential sociodemographic factors that could affect the treatment outcomes of TB patients were also recommended.

## 1. Introduction

Tuberculosis has long been recognized as a major public health problem and leading cause of death alongside with HIV/AIDS [1]. Since then, control efforts including directly observed treatment short course (DOTS) have been initiated by WHO as a strategy [2]. After its launch, DOTS was recommended by international tuberculosis authorities. DOTS has shown to be effective in achieving a high successful treatment outcome [3] and became an important indicator to evaluate the effectiveness of tuberculosis control programs [4].

Early diagnosis and appropriate treatment of TB are among the cornerstones of the DOTS strategy being implemented globally. The targets to control the global epidemic, as recognized by the WHO/Stop TB Partnership and included in the Millennium Development Goals (MDGs), are to diagnose at least 70% of infectious cases and successfully treat at least 85% of them [2].

TB fueled by HIV/AIDS epidemic still remained a major health problem in Ethiopia [5]. According to the 2010/11 national population based survey of Ethiopia, the prevalence of all forms of TB was 224 per 100,000 population [6]. With this prevalence, Ethiopia ranked 7th among the 22 high TB burden countries. In response to this burden, Ethiopia has adopted DOTS as a strategy for TB prevention and control program in the early 1990s [5]. Although DOTS has been estimated to have 100% geographical coverage, it is found only at 95% at health facilities [7].

Studies conducted in southern Ethiopia indicated that DOTS improved the treatment outcomes of tuberculosis and service coverage [8], hence preventing new infections and emergence of drug resistance, but, in Debre Tabor, even though DOTS has been implemented two decades before, the outcomes of treatment and associated factors were not yet assessed. Therefore, this study was aimed to assess the treatment outcomes and associated factors among tuberculosis patients in Debre Tabor, northwest Ethiopia, for the period covering from May 2008 to April 2013.

## 2. Methods

### 2.1. Study Design and Setting

A retrospective study was employed to assess the treatment outcome and associated factors among all forms of TB patients in Debre Tabor. Debre Tabor is a zonal town, 667 kilometers away from the capital, Addis Ababa. The health service delivering institutions are comprised of one general hospital, three health centers, and two private clinics during data collection. Debre Tabor Health Center was randomly selected for this study. Health centers are primary healthcare units capable of serving 15,000–25,000 population.

### 2.2. Data Collection

Data were collected by nurses using a structured sheet specially designed for this study. Patients diagnosed for TB were transferred to DOTS clinic and started treatment according to the national guideline. Patient data on age, sex, residence, type of TB, smear results at baseline and 2nd, 5th, and 7th months, HIV status, year of treatment, patient category during the start of treatment, and the outcomes of treatment were extracted from Debre Tabor Health Center, DOTS clinic.

### 2.3. Inclusion and Exclusion Criteria

Patients diagnosed for any form of TB and started treatment from May 2008 to April 2013 were included while patients under treatment and with incomplete sociodemographic information were excluded.

### 2.4. Laboratory Diagnosis of TB

In Debre Tabor Health Center, pulmonary TB was diagnosed using suggestive clinical signs and symptoms in combination with Ziehl-Neelsen staining and/or chest X-ray. Spot-morning-spot sputum was the specimen used to diagnose PTB. EPTB was diagnosed using clinical information supported with radiography, ultrasound, and cytology and/or pathological procedures through patient referral system with nearby hospitals (Debre Tabor, Felege Hiwot, Gamby, and Gondar) and private clinics.

### 2.5. Laboratory Diagnosis of HIV

For the screening of HIV, the nationally recognized test algorithm was employed in the health center according to the manufacturer's instructions and HIV results were obtained from patient registers. KHB (Shanghai Kehua Bio-Engineering Co., Ltd., China) was used as screening and positive test results were repeated with STAT-PACK (Chembio HIV-1/2 STATPAK*™* Assay, CHEMBIO DIAGNOSTIC SYSTEMS, Inc., Medford, NY, USA). Discordant results from KHB and STAT-PACK were defined by the tie-breaker (UNI-GOLD, HIV, Trinity Biotech PLC, Co., Wicklow, Ireland).

### 2.6. Data Analysis

Data were entered and analyzed using Statistical Package for Social Sciences (SPSS) Version 20.0* (IBM SPSS Statistics for Windows, Armonk, NY: IBM Corp., 2011)*. Descriptive statistics were used to generate and summarize frequencies. Bivariate and multivariate logistic regressions were used to assess the relationship between treatment outcome and independent variables.

## 3. Case and Treatment Outcome Definitions

Patient category, type of TB, and treatment outcome definitions were used according the National Tuberculosis and Leprosy Control Program Guideline (NTBLCP) [5]:

### 3.1. Patient Category

*New Case.* It is a patient who has never had treatment for TB before or has been on anti-TB treatment less than four weeks.

*Relapse.* It is a patient who has been declared cured or has completed treatment of any form of TB in the past but who reports back and was found to be smear positive.

*Treatment Failure.* It is a patient who while on treatment remained smear positive or became again smear positive at the end of the five months or later, after commencing treatment.

*Default.* It is a patient who had previously registered as defaulted from treatment and returns to the health facility and found to be smear positive sputum.

*Transfer In.* It is a patient who started treatment in one health facility (reporting unit) and transferred to another health facility (receiving unit) to continue treatment.

*Unknown.* It is a patient whose category at the start of treatment was not mentioned or neither of the above.

### 3.2. Type of TB

*Smear Positive Pulmonary TB.* It is a patient with at least two sputum specimens positive for AFB by microscopy or one positive sputum specimen for AFB by microscopy and a positive culture or a patient with one positive sputum specimen by microscopy and abnormal chest X-ray indicative of active TB as decided by a clinician.

*Smear Negative Pulmonary TB.* It is a patient with symptoms that are suggestive of TB with three negative sputum smear results by direct microscopy and that do not respond to courses of broad-spectrum antibiotics or three negative sputum smear results by direct microscopy and radiographic abnormalities indicative for pulmonary TB or a patient with three negative sputum smear results by direct microscopy and positive sputum culture for* MTB*.

*Extrapulmonary TB (EPTB).* It is tuberculosis of organs/tissues other than lungs proven by culture, histopathology, and symptoms suggestive of active extrapulmonary TB and decisions made by clinicians to treat with anti-TB drugs. Sputum examination and chest radiographs were used to evaluate the involvement of lungs.

### 3.3. Treatment Outcome

*Cured.* They are patients who completed treatment with negative bacteriology result at the end of treatment.

*Completed.* They are patients who finished treatment, but without bacteriology result at the end of treatment.

*Failure.* They are patients who remained smear positive at five months/later despite correct intake of medication.

*Defaulted.* They are patients who interrupted their treatment after registration for treatment.

*Died.* They are patients who died from any cause during the course of treatment.

*Transferred Out.* They are patients in whom information on treatment outcome cannot be obtained due to transfer to another health facility.

*Successful Treatment.* It is a patient who was cured or has completed treatment.

*Treatment Success Rate (TSR).* It is the sum of the percentages of cured and patients who completed treatment.

## 4. Results

### 4.1. Sociodemographic Characteristics of Patients

Out of 339 TB patients included in this study, 197 (58.1%) were males and 142 (41.9%) were females. Majority (60.8%) of the patients were urban residents. Most cases reported were from 25 to 44 years of age. The mean age and standard deviation (SD) of the patients were 34.9 ± 17.4 (range 1–78) years (Table 1).

### 4.2. Category of Patients

Among TB patients at the start of treatment, 89.1% were new cases, 3.8% were relapsed, 0.6% were failed, 5.3% were transferred in, and 1.2% were unknown cases. Based on the type of TB, 38.9% of the patients were diagnosed as EPTB, 33.3% smear negative PTB, and 27.7% smear positive PTB. Females, patients ≤ 14 years of age, and new TB patients had higher rate of EPTB than smear negative and smear positive PTB. HIV test was done for 71.68% of patients and 12.7% were positive. The rate of TB-HIV coinfection was 16.28% among smear positive PTB, 32.56% among smear negative PTB, and 51.16% among EPTB (Table 2).

**Figure 1:**
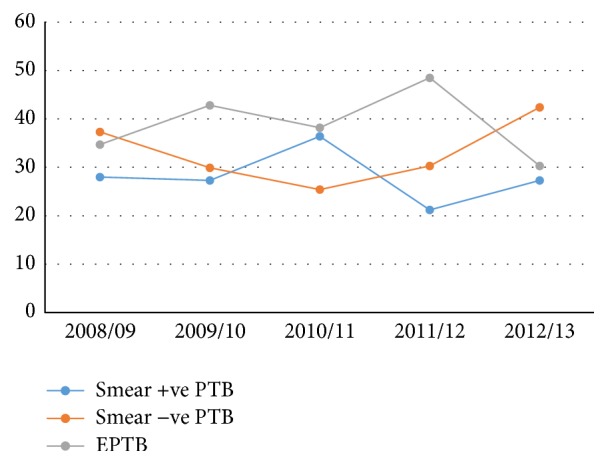
Trends of types of TB over the course of five years at Debre Tabor Health Center, 2008–2013.

Over the five years, the types of TB showed a different trending pattern from one year to another as shown in Figure 1.

### 4.3. Treatment Outcome

Among the TB patients included in this study, 67 patients (19.8%) were cured, 197 (58.1%) completed the treatment, 12 (3.5%) failed, 8 (2.4%) defaulted, 19 (5.6%) died, and 36 (10.6%) patients were transferred out (transferred to another health facility). The rate of cure among all forms TB cases was 19.8% while the rate of treatment completion was 58.1%. The rate of treatment failure, default, and death was 3.5%, 2.4%, and 5.6%, respectively. As age of the patient increased, the trend of completing treatment showed a decreasing pattern while death rate showed an increasing pattern (Table 3).

**Figure 2:**
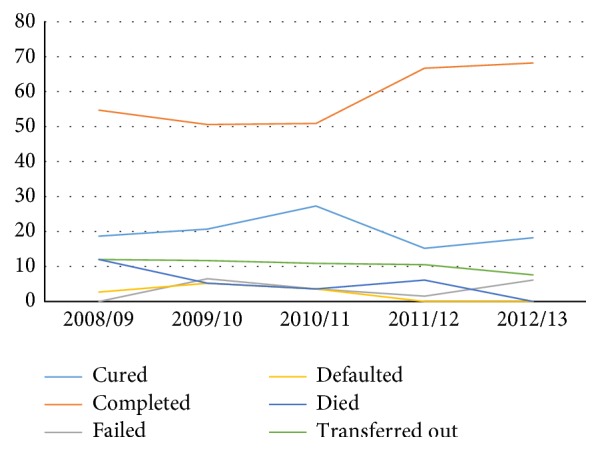
Trends of treatment outcomes of TB over the course of five years at Debre Tabor Health Center.

The trend of transferred-out TB patients has showed a similar pattern while other treatment outcomes had different trends over the course of five years (Figure 2).

Logistic regression was employed to assess sociodemographic variables including age, sex, place of residence, HIV status, baseline smear result, smear result at the 2nd, 5th, and 7th months of treatment, type of TB, year of treatment, and patient category at start of treatment. In the multivariate analysis, the treatment outcome was varied with age, sex, place of resident, and smear result at 2nd month and year of treatment. The odds of successful treatment outcome was 3.807 (95% CI: 1.155–12.544) times higher among patients older than 45 years of age compared to patients younger than 14 years and 25–44 years of age. Females had lower rates of successful treatment (AOR: 0.347, 95% CI: 0.132–0.917).

Patients were less likely to have successful treatment if they were rural residents (AOR: 0.342, 95% CI: 0.118–0.986) compared to urban residents. Successful treatment outcome was less frequent (AOR: 0.056, 95% CI: 0.005–0.577) among smear negative patients than smear positive patients at the 2nd month of treatment.

## 5. Discussion 

Evaluation of the treatment outcome and associated factors for TB patients has the greatest importance in assessing the effectiveness of DOTS program in a country (Table 4). Since the treatment outcome of transferred-out cases was unknown, they were excluded from the final evaluation. Of the 303 patients assessed for their treatment outcomes at Debre Tabor Health Center under DOTS clinic, 264 (87.1%) had successful treatment outcome. This overall treatment success rate for all cases of tuberculosis in our study was supported by various studies conducted in Ethiopia with success rates of 86.2% in northeastern Ethiopia [9] and 85% at Kola Diba [10] while it was higher than studies conducted at Felege Hiwot Referral Hospital (26%) [11], in southern region (49.5%) [8], in Addis Ababa (82.7%) [12], and lower than studies conducted at Enfranz (94.8%) [13]. This satisfactory treatment success rate might be attributable to relatively lower transferred-out rates (10.62%), failures (3.5%), default rate (2.4%), and death rate (5.6%).

Our study revealed that males were more likely to default, to fail, to die, and to transfer out than females and this was consistent with a study conducted in southern Ethiopia [14]. Literatures showed that poor treatment outcomes are associated with inadequate treatment adherence. A study conducted somewhere else in Ethiopia reported that patient behavior and attitude about the disease are major factors affecting adherence to TB treatment [15]. The higher social interaction outside home by males, social isolation associated with TB leading to treatment rejection, alcoholism, and other related behaviors among males might contribute to their higher default, failure, death, and transfer-out rates.

From May 2008 to April 2011, the trends of smear negative PTB showed a decreasing pattern while it showed an increasing trend in later years. Smear negative PTB patients had high treatment success rates compared to EPTB and smear positive PTB patients with treatment success rate of 89.5%, 87.2%, and 84.0%, respectively. The treatment success of smear positive pulmonary TB patients in this study was slightly lower than the 87% WHO international target, 89.0% in Tigray region [16], and 89.3% in the southern region of Ethiopia [17] but higher than studies conducted in Ethiopia including 72.2% in Gambela region [18], 74.8% in southern region [14], and 29.5% in Gondar [3].

The relatively lower overall successful treatment outcome of smear positive PTB in our study when compared to WHO target and other studies conducted in Ethiopia could be attributed to poor smear microscopy resulting in false smear negative PTB. Another possible reason for the relatively lower successful treatment outcome could be lack of tracing out of the treatment outcomes of defaulted patients.

The overall TB-HIV coinfection rate at Debre Tabor Health Center was 12.7%. This figure was lower than previous studies conducted at Gondar University Hospital and northeastern parts of Ethiopia showing high proportions (52.1% and 24.3%, resp.) of coinfection [9, 19]. The lower rate in this study might be explained in terms of unavailability of HIV counseling and testing services in earlier years in the study area or refusal of patients to be tested for HIV (83 patients were registered with their HIV status unknown).

Majority of study participants showing TB-HIV coinfection were those with EPTB (51.2%) followed by smear negative pulmonary (32.6%) and the least coinfection was reported among smear positive pulmonary TB patients (16.3%). The rate of occurrence of TB-HIV coinfection among EPTB patients was three times higher than smear positive pulmonary patients and 1.5 times higher than smear negative PTB. This finding was in agreement with studies conducted in Ethiopia and India [19–21] and evidenced by other literatures as EPTB and smear negative PTB are HIV associated infections.

Contrary to our findings, studies conducted in northeastern part of Ethiopia showed that TB-HIV coinfection was higher among smear positive PTB than smear negative PTB and EPTB [9]. Smear positive PTB and HIV positive patients had experienced higher rate of treatment failure than their counterparts. HIV status, baseline smear result, smear result at the 5th and 7th months of treatment, type of TB, year of treatment, and patient category at start of treatment had no significant association with treatment outcome.

This study also compared the treatment success rates at Debre Tabor Health Center with the national success rates. To this end, there was a relatively similar treatment success rate among patients in Debre Tabor Health Center compared to the national treatment success rates (83.3% versus 84% and 80.9% versus 84%, resp.) in the first two years of treatment while relatively higher success rates were observed in the last three years of treatment (87.8% versus 83%, 91.5% versus 86% and 93.4% versus 91%), respectively (Table 5).

## 6. Conclusion

The treatment outcome of all forms of tuberculosis patients in Debre Tabor was satisfactory as expected from effective implementation of DOTS. Although the observed successful treatment outcome was in agreement with the national target, follow-up of patients during the course of treatment to trace the treatment outcomes of transferred out patients and assessment of other potential sociodemographic factors that could affect the treatment outcomes of TB patients were also recommended.

## Figures and Tables

**Table 1:** Characteristics of TB patients (*N* = 339), Debre Tabor Health Center, 2008–2013.

Characteristics	Frequency	Percentage
Sex		
Male	197	58.1
Female	142	41.9
Residence		
Urban	206	60.8
Rural	133	39.2
Age		
≤24	102	30.1
25–44	140	41.3
≥45	97	28.6
HIV status		
Positive	43	12.7
Negative	200	59.0
Unknown	96	28.3
Patient category		
New	302	89.1
Relapse	13	3.8
Failure	2	0.6
Transfer in	18	5.3
Unknown	4	1.2
Baseline smear result		
Smear positive	94	27.7
Smear negative	285	72.3

**Table 2:** Patients characteristics and type of TB at Debre Tabor Health Center, 2008–2013.

Variables	Type of TB			
	Smear positive PTB (%)	Smear negative PTB (%)	EPTB (%)	Total (%)
Sex				
Male	55 (28.0)	71 (36.0)	71 (36.0)	197 (58.1)
Female	39 (27.5)	42 (29.6)	61 (43.0)	142 (41.9)
Residence				
Urban	52 (25.2)	70 (34)	84 (40.8)	206 (60.8)
Rural	42 (31.6)	43 (32.3)	48 (36.1)	133 (39.2)
Age group				
≤24	24 (23.5)	31 (30.4)	47 (46.1)	102 (30.1)
25–44	49 (35.0)	42 (30.0)	49 (35.0)	140 (41.3)
≥45	21 (21.6)	40 (41.2)	36 (37.1)	97 (28.6)
HIV status				
Positive	7 (16.3)	14 (32.6)	22 (51.2)	43 (12.7)
Negative	60 (30.0)	63 (31.5)	77 (38.5)	200 (59.0)
Unknown	27 (28.1)	36 (37.5)	33 (34.4)	96 (28.3)
Patient category				
New	80 (26.5)	101 (33.4)	121 (40.1)	302 (89.1)
Relapse	11 (84.6)	2 (15.4)	0 (00.0)	13 (3.8)
Failure	2 (100%)	0 (00.0)	0 (00.0)	2 (0.6)
Transferred in	1 (5.6)	9 (50.0)	8 (44.4)	18 (5.3)
Unknown	0 (00.0)	1 (25.0)	3 (75.0)	4 (1.2)
Total	94 (27.7)	113 (33.3)	132 (38.9)	339 (100)

**Table 3:** Characteristics and treatment outcome of TB patients at Debre Tabor, 2008–2013.

Variables	Treatment outcome					
	Cured (%)	Completed (%)	Failed (%)	Defaulted (%)	Died (%)	Transferred out (%)
Sex						
Male	37 (18.8)	103 (52.3)	9 (4.6)	6 (3.0)	14 (7.1)	28 (14.2)
Female	30 (21.1)	94 (66.2)	3 (2.1)	2 (1.4)	5 (3.5)	8 (5.6)
Residence						
Urban	37 (18.0)	121 (58.7)	8 (3.9)	6 (3.0)	15 (7.3)	19 (9.2)
Rural	30 (22.6)	76 (57.1)	4 (3.0)	2 (1.5)	4 (3.0)	17 (12.8)
Age						
≤24	17 (16.7)	63 (61.8)	4 (3.9)	3 (2.9)	1 (1.0)	14 (13.7)
25–44	38 (27.1)	80 (57.1)	3 (2.1)	2 (1.4)	7 (5.0)	10 (7.1)
≥45	12 (12.4)	54 (55.7)	5 (5.2)	3 (3.1)	11 (11.3)	12 (12.4)
Type of TB						
Smear positive PTB	65 (69.1)	3 (3.2)	10 (10.6)	1 (1.1)	2 (2.1)	13 (13.8)
Smear negative PTB	1 (0.9)	93 (82.3)	1 (0.9)	0 (0.0)	10 (8.8)	8 (7.1)
Extrapulmonary TB	1 (0.8)	101 (76.5)	1 (0.8)	7 (5.3)	7 (5.3)	15 (11.4)
HIV status						
Positive	5 (11.6)	30 (69.8)	2 (4.7)	2 (4.7)	2 (4.7)	2 (4.7)
Negative	43 (21.5)	117 (58.5)	9 (4.5)	3 (1.5)	7 (3.5)	21 (10.5)
Unknown	19 (19.8)	50 (52.1)	1 (1.0)	3 (3.1)	10 (10.4)	13 (13.5)
Patient category at start						
New	59 (19.5)	175 (57.9)	8 (2.6)	8 (2.6)	17 (5.6)	35 (11.6)
Relapse	6 (46.2)	2 (15.4)	3 (23.1)	0 (0.0)	2 (15.4)	0 (0.0)
Failure	1 (50.0)	0 (0.0)	1 (50.0)	0 (0.0)	0 (0.0)	0 (0.0)
Transferred in	1 (5.6)	16 (88.9)	0 (0.0)	0 (0.0)	0 (0.0)	1 (5.6)
Unknown	0 (0.0)	4 (100.0)	0 (0.0)	0 (0.0)	0 (0.0)	0 (0.0)
Total	67 (19.8)	197 (58.1)	12 (3.5)	8 (2.4)	19 (5.6)	36 (10.6)

**Table 4:** Factors associated with treatment outcome of TB patients in Debre Tabor, 2008–2013, *N* = 303.

Variables	Treatment outcome		COR (95% CI)	*P* value	AOR (95% CI)	*P* value
	Successful (%)	Unsuccessful (%)				
Sex						
Male	144 (83.2)	29 (16.8)	1.00		1.00	
Female	120 (92.3)	10 (7.7)	0.414 (0.194–0.883)	0.023	0.347 (0.132–0.917)	0.033
Residence						
Urban	158 (85.0)	29 (15.0)	1.00		1.00	
Rural	106 (84.1)	10 (7.9)	0.514 (0.240–1.099)	0.086	0.342 (0.118–0.986)	0.047
Age in groups						
≤24	80 (90.9)	8 (9.1)	1.00		1.00	
25–44	118 (90.8)	12 (9.2)	1.017 (0.398–2.600)	0.972	1.041 (0.298–3.634)	0.949
≥45	66 (77.6)	19 (22.4)	2.879 (1.185–6.996)	0.020	3.807 (1.155–12.544)	0.028
Type of TB						
Smear positive PTB	68 (84.0)	13 (16.0)	1.00		1.00	
Smear negative PTB	94 (89.5)	11 (10.5)	0.612 (0.259–1.449)	0.264	0.240 (0.034–1.724)	0.156
Extrapulmonary TB	102 (87.2)	15 (12.8)	0.796 (0.344–1.718)	0.522	0.355 (0.049–2.603)	0.308
HIV status						
Negative	160 (89.4)	19 (10.6)	1.00		1.00	
Positive	35 (85.4)	6 (14.6)	1.444 (0.537–3.877)	0.466	1.155 (0.322–4.146)	0.825
Unknown	69 (83.1)	14 (16.9)	1.709 (0.810–3.602)	0.159	1.558 (0.312–7.769	0.589
Patient category						
New	234 (87.6)	33 (12.4)	1.00		1.00	
Relapse	8 (61.5)	5 (38.5)	4.432 (1.368–14.355)	0.013	3.906 (0.470–32.483)	0.207
Failure	1 (50.0)	1 (50.0)	7.091 (0.433–116.103)	0.170		
Transferred in	17 (100.0)	0 (0.00)				
Unknown	4 (100.0)	0 (0.00)				
Baseline smear result						
Smear positive	68 (84.0)	13 (16.0)	1.0			
Smear negative	196 (88.3)	26 (12.9)	0.694 (0.338–1.426)	0.320	0.460 (0.071–2.963)	0.414
Smear result at 2nd month						
Positive	3 (30.0)	7 (70.0)	1.00		1.00	
Negative	63 (94.0)	4 (6.0)	0.027 (0.005–0.147)	0.000	0.056 (0.005–0.577)	0.015
Not done	198 (87.6)	28 (12.4)	0.061 (0.015–0.248)	0.000	0.140 (0.004–5.559)	0.296
Year of treatment						
May 2008–April 2009	55 (83.3)	11 (16.7)	2.850 (0.856–9.489)	0.088	2.726 (0.319–23.266)	0.359
May 2009–April 2010	55 (80.9)	13 (19.1)	3.368 (1.035–10.965)	0.044	3.650 (0.806–16.522)	0.093
May 2010–April 2011	43 (87.8)	6 (12.2)	1.988 (0.528–7.485)	0.310	3.624 (0.701–18.727)	0.124
May 2011–April 2012	54 (91.5)	5 (8.5)	1.319 (0.336–5.174)	0.691	1.637 (0.302–8.880)	0.568
May 2012–April 2013	57 (93.4)	4 (6.6)	1.00		1.00	
Total	264 (87.1)	39 (12.9)				

**Table 5:** Comparison of TSR in Debre Tabor with the national TSR, 2008–2013, *N* = 303.

Year of treatments	Treatment success		TSR in DTHC	National TSR
	Success (*N*)	Unsuccessful (*N*)		
May 2008–April 2009	55	11	83.3	84^a^
May 2009–April 2010	55	13	80.9	84^b^
May 2010–April 2011	43	6	87.8	83^c^
May 2011–April 2012	54	5	91.5	86^d^
May 2012–April 2013	57	4	93.4	91^e^

Total	264	39	87.1	

a: WHO 2010, b: WHO 11, c: WHO 2012, d: WHO 2013, and e: WHO 2014.

## Additional Points

*Limitations of the Study*. Patient registration books reviewed lacked data about the HIV status of all TB patients for the year 2008/2009 and few patients for the year 2009/2010 in which patients HIV status was not reported. This will affect the TB-HIV coinfection rate and their treatment outcomes. The retrospective nature of the study could not identify sociodemographic factors that could affect the treatment outcomes of patients that were not included during registration of patients for treatment.

## References

[1] World Health Organization (WHO)

[2] World Health Organization (WHO) (2008). Global tuberculosis control: surveillance, planning and financing. *WHO Report*.

[3] Tessema B., Muche A., Bekele A., Reissig D., Emmrich F., Sack U. (2009). Treatment outcome of tuberculosis patients at Gondar University Teaching Hospital, Northwest Ethiopia. A five-year retrospective study. *BMC Public Health*.

[4] Tessema B., Beer J., Emmrich F., Sack U., Rodloff A. C. (2012). Analysis of gene mutations associated with isoniazid, rifampicin and ethambutol resistance among Mycobacterium tuberculosis isolates from Ethiopia. *BMC Infectious Diseases*.

[5] Ministry of Health of Ethiopia (MOH) (2013). *Guidelines for Clinical and Programmatic Management of TB, TB/HIV and Leprosy*.

[6] Alebachew Z., Kebede A., Tsegaye F. (July 2011). *First Ethiopian National Population Based Tuberculosis Prevalence Survey*.

[7] Federal Ministry of Health: overview of national TB control implementation status.

[8] Shargie E. B., Lindtjørn B. (2005). DOTS improves treatment outcomes and service coverage for tuberculosis in South Ethiopia: a retrospective trend analysis. *BMC Public Health*.

[9] Mekonnen D., Derbie A., Desalegn E. (2015). TB/HIV co-infections and associated factors among patients on directly observed treatment short course in Northeastern Ethiopia: a 4 years retrospective study. *BMC Research Notes*.

[10] Beza M. G., Wubie M. T., Teferi M. D. (2013). A five years tuberculosis treatment outcome at kolla diba health center, dembia district, northwest ethiopia: a retrospective cross-sectional analysis. *Journal of Infectious Diseases and Therapy*.

[11] Biadglegne F., Anagaw B., Debebe T. (2013). A retrospective study on the outcomes of tuberculosis treatment in Felege Hiwot referral hospital, Northwest Ethiopia. *International Journal of Medicine and Medical Sciences*.

[12] Getahun B., Ameni G., Medhin G., Biadgilign S. (2013). Treatment outcome of tuberculosis patients under directly observed treatment in Addis Ababa, Ethiopia. *Brazilian Journal of Infectious Diseases*.

[13] Endris M., Moges F., Belyhun Y., Woldehana E., Esmael A., Unakal C. (2014). Treatment outcome of tuberculosis patients at enfraz health center, Northwest Ethiopia: a five-year retrospective study. *Tuberculosis Research and Treatment*.

[14] Muñoz-Sellart M., Cuevas L. E., Tumato M., Merid Y., Yassin M. A. (2010). Factors associated with poor tuberculosis treatment outcome in the Southern Region of Ethiopia. *International Journal of Tuberculosis and Lung Disease*.

[15] Gelaw M., Genebo T., Dejene A., Lemma E., Eyob G. (2001). Attitude and social consequences of tuberculosis in Addis Ababa, Ethiopia. *East African Medical Journal*.

[16] Berhe G., Enquselassie F., Aseffa A. (2012). Treatment outcome of smear-positive pulmonary tuberculosis patients in Tigray Region, Northern Ethiopia. *BMC Public Health*.

[17] Datiko D. G., Lindtjørn B. (2009). Health extension workers improve tuberculosis case detection and treatment success in southern Ethiopia: a community randomized trial. *PLoS ONE*.

[18] Demeke D., Legesse M., Bati J. (2013). Trend of tuberculosis and treatment outcomes in gambella region with special emphasize on gambella regional hospital, Western Ethiopia. *Mycobacterial Diseases*.

[19] Kassu A., Mengistu G., Ayele B. (2007). Coinfection and clinical manifestations of tuberculosis in human immunodeficiency virus-infected and -uninfected adults at a teaching hospital, Northwest Ethiopia. *Journal of Microbiology, Immunology and Infection*.

[20] Gellete A., Kebede D., Berhane Y. (1997). Tuberculosis and HIV infection in Southern Ethiopia. *The Ethiopian Journal of Health Development*.

[21] Sharma S. K., Mohan A. (2004). Extrapulmonary tuberculosis. *Indian Journal of Medical Research*.

